# The Strength of Weak Ties? Understanding Educational Differences in Parents' Childcare Benefit Knowledge by Applying a Social Capital Approach

**DOI:** 10.1111/1468-4446.70020

**Published:** 2025-08-05

**Authors:** Verena Seibel, Mara Yerkes

**Affiliations:** ^1^ Department of Interdisciplinary Social Science Utrecht University Utrecht the Netherlands; ^2^ Comparative Social Policy in Relation to Social Inequalities Department of Interdisciplinary Social Science Utrecht University Utrecht the Netherlands

## Abstract

Childcare benefits are an important policy instrument to increase the use of formal childcare and often women's participation in the labour market. However, lower‐educated parents continue to make less use of childcare benefits and subsequently less use of formal childcare services. We argue that lower‐educated parents are potentially less knowledgeable about childcare benefit regulations, a knowledge gap that may be explained by educational differences in access to childcare benefit information through parents' social networks. Analysing a representative sample of parents in the Netherlands, we find that lower‐educated parents indeed have less knowledge about childcare benefits than more educated parents. We also find that while there are no educational differences in access to strong ties (e.g., family and friends) and weak ties (e.g., acquaintances and neighbours) as sources of information, lower‐educated parents benefit more from weak ties for knowledge acquisition than intermediate and higher educated parents. We discuss our findings in light of the current debate on the relevance of systemic knowledge about welfare state services for reducing societal inequalities.

## 1 Introduction

Formal childcare is a crucial policy instrument for both parents and children. Formal childcare is important for parents' labour market participation (Boeckmann et al. 2014; Fukkink 2019) and promotes cognitive abilities for children from socio‐economically disadvantaged backgrounds (Drange and Telle 2015; Waldfogel 2006). Yet large discrepancies are visible in the use of formal childcare between groups. Families with lower educational backgrounds make significantly less use of formal childcare (Boeckmann et al. 2014; Roeters and Bucx 2016; Yerkes and Javornik 2019), creating long‐term inequalities for both children and parents (Bonoli et al. 2017).

In countries with market‐based childcare systems, such as the Netherlands, childcare is privatized and costly (Knijn and Lewis 2017). Childcare benefits are, therefore, a crucial policy instrument to facilitate access to formal childcare (Roeters and Bucx 2018; Yerkes and Javornik 2019). A key but understudied factor behind variation in formal childcare use is parents' knowledge of childcare benefits, particularly their eligibility for such benefits. Hummel et al. find that lower‐educated parents perceive childcare benefits to be less accessible than higher educated parents (Hummel et al. 2023), suggesting that lower perceived accessibility may be explained by an absence of knowledge about childcare benefits. Previous studies have indeed found that parents with disadvantaged backgrounds are often insufficiently informed or even unaware of the availability and costs of formal childcare (Roeters and Bucx 2016, 2018) as well as their rights to access formal childcare (Renema 2018; Seibel 2021).

Despite numerous studies on variation in childcare use, an in‐depth analysis of parents' knowledge about childcare benefits and the requirements for accessing these benefits remains largely absent. However, a thorough understanding of parents' knowledge of childcare benefits is crucial: a lack of childcare benefit knowledge among lower‐educated parents can contribute to existing ‘Matthew effects’ found in childcare. In many countries, middle and upper‐class families benefit the most from government spending on formal childcare, and lower‐income families the least, widening existing socio‐economic inequalities between these groups (Pavolini and Van Lancker 2018). Matthew effects in childcare use likely reflect underlying gaps in knowledge about formal childcare opportunities, such as eligibility for childcare benefits. These knowledge gaps can be created or maintained by the sources of information that parents have available to them.

It is well established that social networks are crucial informational resources (Granovetter 1973; Wang et al. 2019). Seibel (2021) demonstrated that these networks also play an important role in explaining childcare knowledge gaps among migrant parents. However, a systematic analysis of the role of social networks in childcare benefit knowledge is missing, mainly due to a lack of suitable data. We extend the childcare literature by addressing this empirical gap, using a probability‐based sample with representative data for the Netherlands to analyse educational differences in parents' knowledge of their eligibility for childcare benefits (hereafter, childcare benefit knowledge) in relation to their social network. We contribute theoretically by using Granovetter's (1973) well‐established distinction between strong and weak ties to examine the extent to which social networks explain potential gaps in parents' childcare benefit knowledge. We look at both parents as well as people who expect to become parents in the coming year, as many formal childcare decisions are taken before a child is even born. Hence, when we refer to parents, we also include expecting parents. By studying how social networks differ among parents of varying educational backgrounds, potentially directly and indirectly affecting their childcare benefit knowledge, we contribute to conceptual debates on barriers to childcare access. We also explore whether access to social networks affects childcare benefit knowledge differently for parents from lower versus higher educational backgrounds.

## 2 Childcare Benefit Regulations in the Netherlands

The Netherlands has a market‐based system of childcare provision, whereby formal childcare consists of relatively expensive, private services. These costs are partially compensated for by the government through childcare benefits provided by the Tax Authority (Yerkes and Javornik 2019). Private childcare services are available for children aged 10 weeks and older. The partial compensation of childcare costs by the government was formalized in the 2005 Child Care Act, intended to create demand‐based childcare services, whereby parents choose the childcare provider and are reimbursed through the tax system. The amount of childcare benefits parents receive is income‐dependent. Most parents are reimbursed for approximately one‐third of childcare costs, but low‐income parents can receive benefits covering up to 90% of actual costs (Akgündüz and Plantenga 2018). Childcare benefits are therefore a crucial policy instrument to facilitate access to otherwise costly formal childcare services for lower‐educated parents in the Netherlands. Yet, only 37% of low‐income households eligible for childcare benefits apply for them (Statistics Netherlands 2021).

For two‐parent families, eligibility for childcare benefits is limited to parents who are *both* in paid employment. Formal childcare in the Netherlands is thus primarily perceived as a policy instrument to enable parents' (and mostly mothers') labour market participation (Akgündüz and Plantenga 2018). There are, however, some very important exceptions: Unemployed parents are entitled to childcare benefits if they are: (a) in education, a labour market re‐integration programme, a civic integration course, or training programme; or (b) have been unemployed for less than 3 months. These exceptions are particularly relevant for lower‐educated parents, who face a higher risk of unemployment (OECD 2023) and are therefore also more likely to (need to) take part in reintegration or training programmes (Bloemendal and van Poeije 2009; Lammers and Kok 2021).

Knowledge about these eligibility regulations is crucial for parents' access to childcare benefits, as well as for parents' employment. For example, if parents are unaware that they are entitled to childcare benefits during unemployment (while following a re‐integration trajectory, e.g.), then parents of this target group may forego formal childcare use, given a fear of high childcare costs. Similarly, unemployed parents with insufficient childcare benefit knowledge may decide to forego participating in a re‐integration programme alltogether if they feel childcare services would be needed to facilitate their participation. Consequently, an absence of childcare benefit knowledge might affect parents' chances of finding a job and thus have potentially longer‐term negative financial consequences for their household.

## 3 Theoretical Background

Research in various domains has shown that knowledge gaps between educational groups are persistent and impactful. For example, lower‐educated individuals tend to have less political knowledge than their higher‐educated counterparts (Fraile 2013; Grönlund and Milner 2006). Similarly, they often possess lower health literacy skills (Jansen et al. 2018; Van Der Heide et al. 2013), which negatively affects their health‐seeking behaviour (Shahid et al. 2022). Knowledge gaps are also evident among high school students, where limited understanding of the educational system is found to undermine their academic performance (Geven and Zwier 2025).

Research on the relationship between education and childcare benefit knowledge is much more limited, but it suggests that education is likely a crucial factor in explaining knowledge gaps. To start, education is a crucial component of *human* capital and thus an indicator of parents' ‘ability to understand the complexity of formal childcare organization and the conditions under which they are eligible to access formal childcare’ (Kingston et al. 2003; Seibel 2021, 1186). Education can also matter for parents' interest in wanting to understand childcare benefits (Andreß and Heien 2001). As higher‐educated parents are more likely to use formal childcare, they are also more likely than lower‐educated parents to have self‐interest in the topic of childcare benefits, thus shaping parents' attempts to acquire childcare benefit knowledge (Seibel 2021; Seibel and Hedegaard 2017). Qualitative findings indeed suggest that lower‐educated parents are less informed than higher‐educated parents about childcare‐related issues, such as financing (Vesely 2013). Moreover, recent empirical evidence suggests educational gaps exist in parents' abilities to navigate childcare benefits, particularly when requiring digital skills (Zamanbin et al. 2025).

A small but growing area of childcare research further suggests education is particularly important for childcare benefit knowledge among vulnerable groups. Mothers with a college degree are better informed about childcare characteristics such as opening hours and the ethnic composition of childcare groups (Camehl et al. 2018), and among migrant populations, lower‐educated parents know significantly less about their childcare rights than higher‐educated parents (Seibel 2021). Based on this empirical evidence, we hypothesize that parents' education is positively associated with their childcare benefit knowledge (H1).

### 3.1 Social Networks and the Strength of Weak Ties

Although education likely functions as an important source of capital (Bourdieu 1986) shaping parents' childcare benefit knowledge, viewing individuals relationally could help to better understand childcare benefit knowledge gaps. Individuals create and maintain bonds with multiple people across the life course. These social networks (i.e., shifting and porous groups of connected individuals) provide individuals with even more resources in the form of social capital (i.e., resources and information gained through personal connections; Coleman 1988) and cultural capital (i.e., non‐economic resources that shape individuals' abilities to navigate institutional structures by ‘knowing the rules of the game’) (Lareau 2015; Lareau and Weininger 2003; Van der Waal et al. 2024). These resources provided by social networks can take on multiple forms, ranging from emotional support or instrumental support to informational support (House et al. 1988). For example, studies in other welfare areas have emphasized the relevance of knowledge as a form of capital for accessing institutional resources, such as in the educational sector (Geven and Zwier 2025) or the healthcare sector (Madden 2015; Paccoud et al. 2020). Applying this idea to childcare benefit knowledge, parents' knowledge can be directly linked to informational support and the extent to which people within one's social network are able to provide information about formal childcare (e.g., who is eligible for childcare benefits and under which conditions).

Evidence suggests social networks are a crucial informational source for parents regarding family policies. Preliminary results from Bartova (2022) show that the presence of a young child in the respondents' immediate social network is an important predictor of better family policy knowledge. Similarly, a study in Norway shows that parents acquire information about the costs and benefits of paternal leave policies mainly via their social network (Dahl et al. 2014). Welteke and Wrohlich (2019) also find that women tend to take similar parental leave as their co‐workers, suggesting, next to a role model effect, that information is transmitted via these networks about the legal possibilities as well as workplace norms. Similar results were also found in Sweden (Carlsson and Reshid 2022). Thus, social networks have the potential to help parents obtain the knowledge necessary to decode bureaucratic language, understand eligibility criteria, and navigate the complex administrative processes associated with accessing childcare benefits, thereby enabling parents' agency and helping them to access childcare benefits (Hummel et al. 2023).

Extending from these studies and incorporating social network theory, we suggest that the role social networks play for parents' childcare benefits knowledge is likely to depend on the *type* of network parents have access to when acquiring information. Granovetter's (1973) classic distinction between strong and weak ties suggests that strong ties (i.e., relations characterized by trust and closeness, such as friends and family members) are important for accessing trustful information quickly (Grieco 1987). Weak ties, in contrast, (i.e., loose connections to a set of varying individuals such as colleagues, neighbours, or acquaintances) increase the chance of accessing valuable resources such as information because they serve as local bridges between otherwise unconnected networks (Granovetter 1973). In networks that consist mainly of strong ties, information is likely to become redundant, as participants of the network may already possess very similar knowledge. Weak ties should therefore have an advantage over strong ties in linking individuals to novel and otherwise inaccessible information (Burt 2004; Greenberg and Fernandez 2016; Rajkumar et al. 2022).

Granovetter's concept of weak and strong ties has mainly been tested in the field of labour market integration. Several studies show that weak ties are crucial transmitters of labour market information and thus ease the entry into the labour market (Brown and Konrad 2001; Rajkumar et al. 2022). In the field of childcare, the distinction between weak and strong ties focuses mainly on strong ties (i.e., family members) being providers of informal childcare as an alternative to formal childcare (Dicks et al. 2022), which can be perceived to be costly and of low quality (Van Gameren 2013). Fewer studies consider weak ties, although the above‐mentioned study by Welteke and Wrohlich (2019) shows that women tend to take parental leave in ways similar to their co‐workers. Such behaviour can be viewed as evidence for the relevance of weak ties (i.e., colleagues), although an empirical comparison to strong ties as a source of information was absent in their study.

Given the absence of an application of social network types in the area of childcare benefit knowledge, we draw on theoretical arguments and empirical findings in similar fields, expecting a similar pattern for how parents acquire knowledge. Close friends and family (i.e., strong ties) might be an easy source of information, yet weak ties are more likely to provide new and valuable information about childcare benefits and eligibility. We therefore hypothesize that seeking information on childcare benefits through weak ties is positively associated with childcare benefit knowledge (H2).

### 3.2 Educational Differences in Receiving Information Through Strong and Weak Ties

It is highly plausible that higher and lower‐educated parents differ in the likelihood that they will be able to access strong and weak ties as sources of information. Variation along educational lines can be expected because there are educational differences in people's opportunities to create weak and strong ties. Higher‐educated people are more likely to be employed (Núñez and Livanos 2010), to volunteer (Gesthuizen and Scheepers 2010), and to be active in political and civic organizations (Egerton 2002; Hillygus 2005) than lower‐educated people: all crucial opportunity factors for establishing contacts with so‐called ‘weak’ relations such as colleagues and neighbours. Hence, in reference to parents seeking information about childcare benefits, we assume that higher‐educated parents have more opportunities to create relationships with weak ties and therefore might be more likely to use their weak ties as informational sources. Research indeed shows that people with higher socio‐economic statuses are more likely to possess weak ties within their social network than people in lower socio‐economic positions (Jahani et al. 2023; Pena‐López and Sánchez‐Santos 2017). Social networks of lower‐educated people, on the other hand, are typically more dense and geographically close (Bailey et al. 2018). As a result, less educated people often turn to strong ties (family and close friends) when seeking support (Antonucci et al. 2019). Similarly, research from other socially disadvantaged groups, such as ethnic minorities, shows that parents with few socio‐economic resources particularly tend to seek information from people considered to be strong ties rather than weak ties (Karoly and Gonzalez 2018; Lastikka and Lipponen 2016; Miller et al. 2014; Seibel 2021).

Following these theoretical considerations, whereby weak ties are particularly important for acquiring childcare benefit knowledge, it is likely that higher‐educated parents possess more childcare benefit knowledge because they are more likely to make use of their weak ties when seeking childcare benefit information. We therefore hypothesize that the positive association between education on childcare benefit knowledge is partially mediated by accessing information through weak ties (H3).

### 3.3 Educational Differences in Benefiting From Strong and Weak Ties

Parents may not only differ in their access to specific networks. Parents with higher educational backgrounds might also *benefit differently* from their network compared to parents with lower educational backgrounds. We explore two potential theoretical mechanisms here.

On the one hand, lower‐educated parents might benefit more from their social networks in terms of childcare benefit knowledge than higher‐educated parents. Although higher‐educated parents use their social networks for information seeking, they also rely more on alternative (online) informational sources, such as government or childcare webpages, than lower‐educated parents (Disalvo et al. 2016; Garg and Sengupta 2019), given potentially greater abilities to navigate and understand online information channels (Hummel et al. 2023). Consequently, higher‐educated parents might be less dependent on their social networks for acquiring childcare benefit information, while social networks remain a crucial informational source for lower‐educated parents (Karoly and Gonzalez 2018), who struggle with the navigation of the administrative childcare benefit landscape (Zamanbin et al. 2025). One expectation can therefore be that the association between strong and weak ties and childcare benefit knowledge will be stronger for parents with low educational levels compared to parents with intermediate or higher educational levels (H4a).

On the other hand, theoretical arguments could also lead in the opposite direction. According to the principle of homophily, people tend to seek contact with those who resemble them and are similar in certain characteristics, such as educational level (McPherson et al. 2001). People tend to marry within their educational group (Blossfeld 2009) and seek friendships with those who share similar educational backgrounds (Chetty et al. 2022). Hence, the weak and strong ties of higher‐educated parents most likely consist of other highly educated people, whereas weak and strong ties of lower‐educated parents most likely consist of people with similarly low levels of education.

Research in other areas, like labour market integration research, suggests that these differences in the educational composition of people's networks matter for individual outcomes. For example, high amounts of education within a network increase an individual's labour market integration chances (Lin 2003). Highly educated contacts are assumed to have more information than less educated contacts (Pena‐López and Sánchez‐Santos 2017). For the acquisition of childcare benefit knowledge, we might expect a similar mechanism. The social networks of higher‐educated parents might be more likely to provide correct and relevant information about formal childcare benefits compared to the social networks of lower‐educated parents, simply because higher‐educated people may possess more of the skills needed to navigate social policy and (online) service systems (e.g., Hummel et al. 2023). Based on these theoretical and empirical elaborations, we expect that the association between strong and weak ties and childcare benefit knowledge will be weaker for parents with low educational levels than for parents with intermediate or higher educational levels (H4b).

## 4 Data, Measurements, and Methods

### 4.1 Data

We used data from the study ‘From Perceptions to Behavior? Examining Knowledge Barriers to Formal Childcare among Dutch Parents’, collected in July 2021 within the LISS panel (Longitudinal Internet Studies for the Social Sciences, https://www.lissdata.nl). The LISS panel consists of appromxiametly 5000 households, comprising around 7500 individuals and is a representative, online survey panel based on a true probability sample drawn from population registers by the Dutch National Statistics Office (CBS). LISS panel data collection begaan in 2007; respondents were recruited offline through the postal service. Households without computer or internet access are provided with the necessary equipment to complete the survey. Respondents are compensated financially for each survey participation, whereby the amount depends on the length of the survey.

The sample drawn for the purpose of this study included all panel members who at the time of sampling had at least one child between 0 and 12 years (the age until which childcare benefits can be used in the Netherlands) as well as individuals who potentially could expect to have children within the next 12 months. The latter group contained adults aged 24 to 44 without children. A total of 2033 panel members were eligible for the study; 1439 respondents initially took part (response rate of 70.8%). However, respondents who indicated during the first filter question that they neither had children nor were expecting to have a child within the next 12 months were excluded from the survey (*n* = 567). Most variables in our analyses contained a limited amount of missings (+/− 2), leading to a total survey sample of 863 respondents.

### 4.2 Dependent Variable

Our dependent variable was childcare benefit knowledge: Respondents were presented with six different scenarios, describing a fictitious couple based on their employment status. Respondents were asked to indicate for each scenario whether they thought this couple was eligible for childcare benefits or not (see Supporting Information S1: Table A1 for detailed measurement). Experts in the field were consulted in the creation of the scenarios, with chosen scenarios based on the formal eligibility criteria established under Dutch law. As previously discussed, the most common eligibility criterion is that both parents must be employed. However, childcare benefit regulations allow for three main exceptions to this ‘employment rule’: (1) enrolment in an educational programme; (2) participation in a civic integration course or a reintegration programme; and (3) unemployment for less than 3 months. In particular, the last two regulations target parents with lower levels of education to ensure that they continue or start making use of childcare despite being out of the labour market. The six scenarios presented to respondents capture these eligibility regulations and test parents awareness of them.

Answer categories were: 1 = yes, the couple is entitled to childcare benefits; 2 = the couple is not entitled to childcare benefits; and 3 = I don't know. We were mainly interested in whether parents' childcare benefit knowledge was correct. We therefore combined the category 3 with the incorrect answer (either 1 or 2, depending on the scenario), resulting in a dichotomous variable whereby 1 = ‘provides the correct answer’ and 0 = ‘does not provide the correct answer’ (reference category). Finally, we calculated an overall knowledge score for each parent based on the number of scenarios answered correctly. This score was calculated by taking the share of correct answers as a proportion of the total number of possible correct answers (=6). Hence, respondents who gave one correct answer would score 0.16, respondents who gave two correct answers would score 0.33, and so on.

### 4.3 Independent Variables

Education was measured based on the categorization of Statistics Netherlands (CBS) with 1 = primary school; 2 = intermediate secondary education; 3 = higher secondary education; 4 = intermediate vocational education; 5 = higher vocational education; and 6 = university. Given lower response rates among respondents with primary and secondary education, we regrouped these six categories into three categories reflecting 1 = lower (primary and intermediate secondary education, reference category); 2 = intermediate (higher secondary and intermediate vocational education); and 3 = higher education (tertiary vocational education and university), thereby following the 2011 Dutch International Standard Classification of Education (ISCED).

To measure social capital in relation to childcare benefit knowledge, we asked respondents ‘How likely are you to ask the following people for information about childcare benefits’: (a) Your friends or relatives (e.g., parents, siblings, grandparents, cousins, uncles, aunts; which we operationalize as *strong ties*) and (b) your co‐workers neighbours, or distant acquaintances (which we operationalize as *weak ties*). Answer categories were: 1 = very unlikely; 2 = unlikely; 3 = likely; 4 = very likely; and 5 = don't know. Both variables (strong and weak ties) were recoded into three categories with 1 = not (very) likely; 2= (very) likely; and 3 = don't know. Note that respondents who answered ‘don't know’ on strong ties overlap almost 100% on the weak ties variable. For that reason, the ‘don't know’ category is omitted due to multicollinearity in the estimated models that contain both variables (strong and weak ties). Given the size of the ‘don't know’ categories, we have also included robustness checks (see below).

### 4.4 Control Variables

Given the comparably small sample size we carefully selected our control variables in order to avoid over‐controling the models, following Kohler et al.’s approach of control variable selection (Kohler et al. 2024). We therefore controlled for variables likely to affect both our dependent variable, childcare benefit knowledge, and/or our main independent variables, education and social capital (e.g., Hummel et al. 2023; Roeters and Bucx 2016; Seibel 2021). We control for gender (0 = male; 1 = female), as women vary from men in their social network composition (Moore 1990) and have been found to possess more knowledge about childcare eligibility criteria than men (Seibel 2021), likely given their primary responsibility in caregiving. Age has also been found to correlate with social networks (Bruine de Bruin et al. 2020), as has employment status (0 = not employed; 1 = employed) (Rözer et al. 2020) and marital status (0 = not married (including divorced, widowed, and separated); 1 = married) (Haggerty et al. 2022). Finally, we also control for parenthood. The sample consists of respondents who are already parents (= 1) or expecting to be parents within the next 12 months (= 0). Parenthood impacts people's social networks (Diaz et al. 2011) and we also expect parents to have greater knowledge about childcare benefits than expecting parents. We note that in our robustness checks, we also consider additional control variables such as household income, migration status, and living arrangements (see section ‘robustness checks’ for measurement description).

### 4.5 Analysis

Our analysis proceeded in three steps. In a first step, we descriptively assessed respondents' childcare benefit knowledge and looked at the variation in this knowledge across educational levels. In a second step, we used linear regression with robust standard errors to estimate the association between education and parents' childcare benefit knowledge and its interrelation with the likelihood of accessing information through strong and weak ties. We thereby applied a KHB decomposition analysis (Kohler and Karlson 2022) using STATA to assess whether strong and weak ties mediate the relationship between parents' education and their childcare benefit knowledge. In a third step, we estimated an interaction between parents' education and strong and weak ties to test whether education moderates the relationship between parents' strong and weak ties and their childcare benefit knowledge. Lastly, we conducted several robustness checks to examine the role of potential confounders, such as income, migration background, and living arrangements.

## 5 Results

**FIGURE 1:**
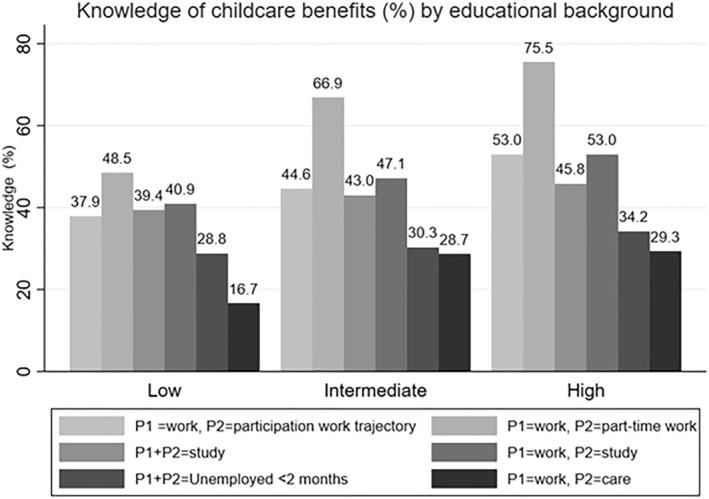
Knowledge of childcare benefits (%) by educational background and eligibility condition. Each bar represents a different eligibility condition, whereby P1 = parent 1 and P2 = parent 2.

Figure 1 shows the distribution of knowledge for each of the eligibility conditions for childcare benefits across low, intermediate, and high education levels for parents. The descriptive results indicate that childcare benefit knowledge differs across parents of varying educational levels and differs across eligibility conditions as well. The educational gap in childcare benefit knowledge is particularly prevalent regarding eligibility related to part‐time work. Whereas more than 75% of higher‐educated respondents are aware that parents who work part‐time are eligible for childcare benefits, this is only the case for 48% of low educated parents.

Descriptive results further show (see Supporting Information S1: Table A2) that approximately 24% of the sample report being (very) likely to receive information about childcare benefits from their so‐called weak ties, and 40% from their strong ties. 10% of the respondents say that they do not know if their social network would be likely to provide childcare benefit information, which can be interpreted as a blurred or limited awareness of the network's informational capacity.

The results of the multivariate analysis are presented in Table 1. Our first hypothesis tests whether higher education is associated with higher childcare benefit knowledge. Model A (Table 1), which contains education and the control variables, partly confirms this hypothesis. Although we find no significant differences between intermediate and lower‐educated parents, parents with higher education know significantly more about childcare benefit eligibility than parents with lower education.

**TABLE 1:** Associations between education, social capital and knowledge about childcare benefits (linear regression, robust standard errors in parentheses).

	Model A	Model B	Model C	Model D	Model E
Educational level					
Low	*Ref.*	*Ref.*	*Ref.*	*Ref.*	*Ref.*
Intermediate	0.050	0.029	0.031	0.029	0.046
	(0.179)	(0.397)	(0.382)	(0.406)	(0.363)
High	0.087*	0.054	0.057	0.055	0.081^a^
	(0.017)	(0.115)	(0.101)	(0.109)	(0.100)
Weak ties					
No information provision		*Ref.*		*Ref.*	*Ref.*
Information provision		0.016		0.031	0.231*
		(0.421)		(0.169)	(0.012)
Don't know		−0.248***		−0.256***	−0.225**
		(0.000)		(0.000)	(0.004)
Strong ties					
No information provision			*Ref.*	*Ref.*	*Ref.*
Information provision			−0.015	−0.029	−0.088
			(0.422)	(0.171)	(0.269)
Don't know			−0.259***	*Omitted*	*Omitted*
			(0.000)		
Interactions					
Weak ties: Information provision					
# Intermediate educated					−0.231*
					(0.021)
#Highly educated					−0.202*
					(0.036)
Weak ties: Don't know					
# Intermediate educated					−0.067
					(0.451)
#Highly educated					0.005
					(0.957)
Strong ties:Information provision					
# Intermediate educated					0.102
					(0.247)
#Highly educated					0.043
					(0.606)
Strong ties: Don't know					
# Intermediate educated					*Omitted*
#Highly educated					*Omitted*
Controls					
Age	−0.006***	−0.006***	−0.006***	−0.006***	−0.006***
	(0.000)	(0.000)	(0.000)	(0.000)	(0.000)
Female	0.038*	0.032^a^	0.030^a^	0.032^a^	0.033^a^
	(0.041)	(0.072)	(0.090)	(0.075)	(0.064)
Employed	0.048^a^	0.038	0.036	0.039	0.040^a^
	(0.057)	(0.112)	(0.130)	(0.104)	(0.085)
Parents	0.052^a^	0.058*	0.053^a^	0.053^a^	0.057*
	(0.076)	(0.039)	(0.065)	(0.062)	(0.045)
Married	−0.010	−0.014	−0.015	−0.015	−0.014
	(0.607)	(0.425)	(0.383)	(0.397)	(0.416)
*R* ^2^	0.0412	0.1214	0.1214	0.1233	0.1312
*N*	863	863	863	863	863

^a^ *p* < 0.10.

* *p* < 0.05.

** *p* < 0.01.

*** *p* < 0.001.

To investigate whether childcare benefit knowledge is associated with social networks, our second hypothesis stated that seeking information on childcare benefits through weak ties would be positively associated with childcare benefit knowledge. Models B and C examine the role of social ties in accessing information, with Model B including weak ties and Model C strong ties. We note two results in particular. First, access to weak or strong ties is not significantly associated with childcare benefit knowledge. In other words: Parents who are likely to seek information about childcare benefits via their strong and weak ties do not have greater knowledge than those who do not, leading us to reject hypothesis 2, where we assumed a positive association between weak ties and childcare benefit knowledge. Interestingly, however, parents who selected ‘don't know’, indicating uncertainty about whether they could access either strong or weak ties, were significantly less knowledgeable about childcare benefits than parents who were able to give a clear answer. We return to this finding in the discussion.

Second, the main estimate of education decreased from model A to model B and to model C and became insignificant, suggesting that access to social capital partially explains educational differences. To examine this further, we first estimated the association between education and the likelihood of accessing strong and weak ties for information acquisition (Table 2). While parents with different education levels are equally likely to report using strong or weak ties, higher‐educated parents are less likely to respond ‘don't know.’ In other words, less educated parents show more uncertainty about whether their networks can provide relevant information. In a second step, we applied a KHB decomposition analysis (Table 3), in which the overall association linked to education was separated into a direct component (education) and an indirect component via social capital across models (Kohler and Karlson 2022). Results indicate that the coefficient change (both from Model A to Model B, and from Model A to Model C) of ‘highly educated’ is indeed significant. Given the results discussed above, we can conclude that this coefficient change was due to the educational differences in *not knowing* about their access to information about childcare benefits, rather than educational differences in accessing weak ties as information sources. We therefore reject hypothesis 3, as the observed association between education and access to information is not accounted for by differences in the use of weak ties, but rather by differences in perceived access to informational network sources.

**TABLE 2:** Association between education and information provision by weak and strong ties (information provision as baseline), multinominal logistic regressions.

	Weak ties		Strong ties	
	No information provision versus information provision	Don't know versus information provision	No information provision versus information provision	Don't know versus information provision
Educational level				
Low	*Ref.*	*Ref.*	*Ref.*	*Ref.*
Intermediate	−0.432	−0.890^a^	−0.096	−0.635
	(0.257)	(0.056)	(0.775)	(0.122)
High	−0.601	−1.581***	−0.410	−1.380***
	(0.108)	(0.001)	(0.211)	(0.001)
Controls				
Age	0.021	0.015	0.058***	0.035^a^
	(0.159)	(0.498)	(0.000)	(0.093)
Female	0.441*	−0.021	0.259	−0.194
	(0.012)	(0.941)	(0.106)	(0.470)
Employed	0.568*	−0.091	0.104	−0.452
	(0.015)	(0.788)	(0.647)	(0.156)
Parents	0.566*	0.581	1.178***	0.608
	(0.028)	(0.178)	(0.000)	(0.136)
Married	0.199	−0.089	0.229	−0.100
	(0.249)	(0.741)	(0.155)	(0.693)
*R* ^2^	0.0305	0.0614	0.0614	0.0614
*N*	863	863	863	863

^a^ *p* < 0.10.

* *p* < 0.05.

**p < 0.01.

*** *p* < 0.001.

**TABLE 3:** KHB decomposition analysis: Significance (*p*‐value) of the indirect association between education and strong and weak ties (controlled for age, gender, employment, parental status, marital status).

	Weak ties as mediator	Strong ties as mediator
Educational level		
Low	*Ref.*	*Ref.*
Intermediate	0.021	0.023
	(0.014)	(0.014)
High	0.031*	0.030*
	(0.015)	(0.014)

^a^ *p* < 0.10.

* *p* < 0.05.

**p < 0.01.

***p < 0.001.

Model D includes both strong and weak ties to examine whether presenting them together alters their associations with the outcome, which would suggest intercorrelation. This is not the case: the main associations remain stable. The ‘don't know’ category of the ‘strong ties’ measurement is omitted in Model D. This is because parents who indicated they do not know if they have access to strong ties for information provision also do not know whether they have access to weak ties.

Model E examines whether education moderates the relationship between access to social ties and childcare benefit knowledge. It includes two interaction effects: one between education and weak ties and one between education and strong ties. Results show a significant interaction for weak ties: While weak ties are positively associated with childcare benefit knowledge overall, this relationship is stronger for lower‐educated parents than for those with intermediate or higher education. This result partially supports Hypothesis 4a, while leading us to reject Hypothesis 4b. In other words: lower‐educated parents benefit more from their weak ties than intermediate or highly educated parents, which is partly in line with hypothesis 4a; hypothesis 4b therefore has to be rejected. The results of Model E help explain why the association between weak ties and knowledge of childcare benefits was small and not statistically significant in Models C and D. One explanation could be that the associations for lower‐educated parents and for intermediate and higher‐educated parents offset each other. Finally, we find that the association between strong ties and childcare benefits knowledge does not differ significantly across the three educational groups.

### 5.1 Robustness Checks

We conducted several robustness checks. First, we included the household net income in the robustness check, which was not included in the main models, given collinearity with educational level. We additionally estimated our models taking into account parents' migration background as a control variable, given that parents with a migration background are known to differ from parents without a migration background in relation to their childcare knowledge (Seibel 2021). However, migrant status was not included in our main analyses as a control variable given a high number of missings (*n* = 124). To ensure the robustness of our models, we re‐ran the main analyses including a control for income and having a migration background. In order to account for the socialization effect, we used the question ‘Which language or languages did you grow up speaking’, with ‘Dutch’ (0) and ‘a language other than Dutch’ (1).

All additional models are presented in the Supporting Information S1 (Table A3). The main results remain the same: Higher educated parents have more knowledge about childcare benefits than lower educated parents. Also, the interaction effect between education and weak ties remains similar in size and significant, though on a lower significance level than in the main models. This is most likely due to the lower sample size caused by including the migration background variable. The variable migration background does impact parents' childcare benefit knowledge: parents who grew up with Dutch in the household (hence, not having a migration background) know more about childcare benefits than parents who grew up with another language at home. Income, however, does not impact parents' knowledge about childcare benefits.

Finally, to account for single parents, we examined whether the models remained the same when controlling for living with a partner rather than marital status. Single parents have been found to have fragile social networks (McArthur and Winkworth 2017), which may hinder their access to knowledge about childcare benefits. However, all results (not presented here) remain unchanged, and partnership is not significantly associated with either childcare benefit knowledge or access to childcare information via social network.

## 6 Conclusion

We contribute to the extensive body of research on barriers to formal childcare use (Nieuwenhuis 2022; Pavolini and Van Lancker 2018; Yerkes and Javornik 2019) by studying a crucial, but thus far neglected aspect, namely, educational differences in parents' childcare benefit knowledge. Our results confirm the existence of an education‐based gap in childcare benefit knowledge, which can contribute to already existing Matthew effects in childcare use (Pavolini and Van Lancker 2018): Parents with less than tertiary levels of education possess less childcare benefit knowledge than tertiary‐educated parents.

We have argued that part of this education gap in childcare benefit knowledge can be explained by educational differences in how parents access their social network for childcare benefit information. We find some support for this idea, but not in the direction we expected. Specifically, we do not find significant educational differences in the reported likelihood of seeking information from strong ties (such as family members and friends) or weak ties (such as neighbours, acquaintances or colleagues). In other words, parents with lower educational levels are, on average, just as likely as highly educated parents to say they would turn to these contacts for information on childcare benefits. However, where educational differences emerge is in the uncertainty about using these ties: Parents with lower levels of education are significantly more likely to select *‘don't know’* when asked whether they would approach their network for childcare benefit information. Importantly, this uncertainty is independent of the strength of the tie. We interpret this not as an indicator of indecision per se, but as an indicator of limited awareness of the informational resources within one's network. This unexpected finding is crucial: If parents are unsure whether their network members can provide relevant information, they may be less likely to mobilize those ties. Indeed, we find that those who answered *‘don't know’* were also significantly less likely to possess accurate knowledge about childcare benefits. This suggests that network ambiguity — the lack of clear awareness about who knows what — constitutes a distinct form of informational disadvantage, and helps explain why parents with lower educational backgrounds are less informed about available childcare benefits.

Finally, we were interested in whether access to information provided by the social network works differently for lower, intermediate, or higher‐educated parents. We find that, for lower‐educated parents, access to weak ties is positively associated with their likelihood of possessing knowledge about childcare benefits. However, intermediate and higher‐educated parents do not benefit from their weak ties in terms of childcare benefit knowledge accumulation. One reason for this finding could be that higher‐educated people are more likely to obtain childcare benefit information from formal channels such as government webpages (Disalvo et al. 2016; Garg and Sengupta 2019). Lower‐educated people run a higher risk of experiencing administrative burden when accessing such formal information sources (Hummel et al. 2023; Martin et al. 2024). Hence, while higher‐educated parents are likely to access both formal and informal information channels, lower educated parents are more dependent on their social networks, particularly their weak ties.

Taken together, these results can provide insights into understudied barriers to childcare use and the consequences for parents. A lack of childcare benefit knowledge can affect parents' decisions not to use formal childcare as well as their decision whether and to what extent to participate in paid work. If parents are only able to participate in paid work if they have access to formal childcare (e.g., because informal care is not possible), *and* parents believe they are only eligible for childcare benefits if they work full‐time, then these parents may not seek employment. Potential reasons for non‐take‐up could stem, among others, from a fear of high childcare costs (Roeters and Bucx 2016, 2018).

We note a number of limitations. Our study does not provide information on how parents evaluate the information they receive. Research about the influence of social networks on parents' school choices shows that parents give priority to information provided by people perceived to have high authority and affinity (Fong 2019). It is likely that this is also the case for childcare benefits. For example, while weak ties may be more likely to provide information about childcare benefits, information from strong ties may be more trusted and therefore more relevant to parents' knowledge acquisition. Lower‐educated people are generally found to trust government institutions less than higher‐educated people (Hakhverdian and Mayne 2012; Kulin and Johansson Sevä 2021) and might, for that reason, rely more on their social networks for information acquisition. We were unable to test this relationship due to a lack of adequate measurements of institutional trust; however, we advise future research to explore this relationship between government attitudes and childcare benefit knowledge further. Also, qualitative research could help shed light on why parents prefer certain networks to others for obtaining trustful information about childcare benefits, particularly for those groups in society who have the least access to formal childcare services. In addition, a social network approach using ego‐centric or whole‐network data would allow an in‐depth exploration of the specific characteristics of informal information sources beyond the weak‐strong tie dichotomy, taking into account people's gender, status, and position within the network.

Limitations aside, the findings provide a foundation for future research into benefit knowledge for studies of family policies. Our results suggest that one potential factor hindering low‐educated families' use of formal childcare is the administrative burden associated with complex application procedures, particularly in market‐led childcare systems (Hummel et al. 2023; Martin et al. 2024). Administrative burden may be particularly relevant in the case of childcare benefits (Zamanbin et al. 2025), but more research is needed to understand how administrative burden is experienced and what this means for childcare benefit knowledge and childcare use. Some parents may feel confronted with the burden of needing multiple types of information to complete application procedures (e.g., in the case of childcare benefits: own and partner income, continuous reporting of any changes in income, own and partner work hours, hourly childcare rates, etc.). Other parents may experience administrative burden accompanied by the fear of making a mistake that can have serious financial consequences if they have to retroactively pay back payments received, something which can be particularly harmful for low‐income families. In the Netherlands in particular, fear of making mistakes in the application for childcare benefits could be very high following a recent childcare benefit crisis, whereby the Dutch Tax Authority falsely accused thousands of families of fraudulently receiving the benefit (Van Dam et al. 2020). From a policy and scholarly perspective, attention to lowering this administrative burden while improving childcare benefit knowledge is crucial.

## Conflicts of Interest

The authors declare no conflicts of interest.

## Supporting information


Supporting Information S1

## Data Availability

The data that support the findings of this study are openly available in the Longitudinal Internet Studies for the Social Sciences (LISS) Panel at https://doi.org/10.17026/dans‐zcp‐my68. Reference number: 253.

## References

[bjos70020-bib-0001] Akgündüz, Y. E. , and J. Plantenga . 2018. “Childcare Prices and Maternal Employment: A meta‐analysis.” Journal of Economic Surveys 32, no. 1: 118–133. 10.1111/JOES.12192.

[bjos70020-bib-0002] Andreß, H. , and T. Heien . 2001. “Four Worlds of Welfare State Attitudes? A Comparison of Germany, Norway, and the United States.” European Sociological Review 17, no. 4: 337–356. 10.1093/esr/17.4.337.

[bjos70020-bib-0003] Antonucci, T. , K. Ajrouch , N. Webster , and L. Zahodne . 2019. “Social Relations Across the Life Span: Scientific Advances, Emerging Issues, and Future Challenges.” Annual Review of Developmental Psychology 1: 313–336. https://www.annualreviews.org/doi/pdf/10.1146/annurev‐devpsych‐121318‐085212.

[bjos70020-bib-0004] Bailey, M. , R. Cao , T. Kuchler , and J. Stroebel . 2018. “The Economic Effects of Social Networks: Evidence From the Housing Market.” Journal of Political Economy 126, no. 6: 2224–2276. 10.1086/700073/ASSET/IMAGES/LARGE/FG6.JPEG.

[bjos70020-bib-0005] Bartova, A. 2022. “Family Policy Awareness Across the Lifecourse.” ESPAnet Annual Conference 2022.

[bjos70020-bib-0006] Bloemendal, C. , and A. van Poeije . 2009. Wie Volgen Een re‐integratietraject? CBS. https://www.cbs.nl/nl‐nl/achtergrond/2009/36/wie‐volgen‐een‐re‐integratietraject.

[bjos70020-bib-0007] Blossfeld, H.‐P. 2009. “Educational Assortative Marriage in Comparative Perspective on JSTOR.” Annual Review of Sociology 35, no. 1: 513–530. https://www.jstor.org/stable/27800089.

[bjos70020-bib-0008] Boeckmann, I. , J. Misra , and M. J. Budig . 2014. “Cultural and Institutional Factors Shaping Mothers’ Employment and Working Hours in Postindustrial Countries.” Social Forces 93, no. 4: 1301–1333. 10.1093/sf/sou119.

[bjos70020-bib-0009] Bonoli, G. , B. Cantillon , and W. Van Lancker . 2017. “Social Investment and the Matthew Effect.” In The Uses of Social Investment, edited by A. Hemerijck , 66–76. Oxford University Press. 10.1093/oso/9780198790488.003.0005.

[bjos70020-bib-0010] Bourdieu, P. 1986. “Forms of Capital.” Cultural Theory: An Anthology 1: 8193. https://www.sciencedirect.com/science/article/pii/S0277953605002546.

[bjos70020-bib-0011] Brown, D. W. , and A. M. Konrad . 2001. “Granovetter Was Right.” Group & Organization Management 26, no. 4: 434–462. 10.1177/1059601101264003.

[bjos70020-bib-0012] Bruine de Bruin, W. , A. M. Parker , and J. N. Strough . 2020. “Age Differences in Reported Social Networks and well‐being.” Psychology and Aging 35, no. 2: 159–168. 10.1037/PAG0000415. 31697096 PMC7122684

[bjos70020-bib-0013] Burt, R. S. 2004. “Structural Holes and Good Ideas.” American Journal of Sociology 110, no. 2: 349–399. 10.1086/421787.

[bjos70020-bib-0014] Camehl, G. F. , P. S. Schober , and C. K. Spiess . 2018. “Information Asymmetries Between Parents and Educators in German Childcare Institutions.” Education Economics 26, no. 6: 624–646. 10.1080/09645292.2018.1463358.

[bjos70020-bib-0015] Carlsson, M. , and A. A. Reshid . 2022. “Co‐Worker Peer Effects on Parental Leave Take‐Up.” Scandinavian Journal of Economics: 1–28. 10.1111/SJOE.12485.

[bjos70020-bib-0016] Chetty, R. , M. O. Jackson , T. Kuchler , et al. 2022. “Social Capital II: Determinants of Economic Connectedness.” Nature 608, no. 7921: 122–134. 10.1038/s41586-022-04997-3. 35915343 PMC9352593

[bjos70020-bib-0017] Coleman, J. S. 1988. “Social Capital in the Creation of Human Capital.” American Journal of Sociology 94: 95–120. 10.1086/228943.

[bjos70020-bib-0018] Dahl, G. B. , K. V. Løken , and M. Mogstad . 2014. “Peer Effects in Program Participation.” American Economic Review 104, no. 7: 2049–2074. 10.1257/AER.104.7.2049.

[bjos70020-bib-0019] Diaz, B. A. , T. Fent , A. Prskawetz , and L. Bernardi . 2011. “Transition to Parenthood: The Role of Social Interaction and Endogenous Networks.” Demography 48, no. 2: 559–579. 10.1007/s13524-011-0023-6. 21523518

[bjos70020-bib-0020] Dicks, A. , M. Levels , R. van der Velden , and M. C. Mills . 2022. “How Young Mothers Rely on Kin Networks and Formal Childcare to Avoid Becoming NEET in the Netherlands.” Frontiers in Sociology 6: 787532. 10.3389/fsoc.2021.787532. 35155664 PMC8829039

[bjos70020-bib-0021] Disalvo, B. , P. K. Roshan , and B. Morrison . 2016. “Information Seeking Practices of Parents: Exploring Skills, Face Threats and Social Networks.” Conference on Human Factors in Computing Systems ‐ Proceedings: 623–634. 10.1145/2858036.2858586.

[bjos70020-bib-0022] Drange, N. , and K. Telle . 2015. “Promoting Integration of Immigrants: Effects of Free Child Care on Child Enrollment and Parental Employment.” Labour Economics 34: 26–38. 10.1016/j.labeco.2015.03.006.

[bjos70020-bib-0023] Egerton, M. 2002. “Higher Education and Civic Engagement.” British Journal of Sociology 53, no. 4: 603–620. 10.1080/0007131022000021506. 12556285

[bjos70020-bib-0024] Fong, K. 2019. “Subject to Evaluation: How Parents Assess and Mobilize Information From Social Networks in School Choice.” Sociological Forum 34, no. 1: 158–180. 10.1111/SOCF.12483.

[bjos70020-bib-0025] Fraile, M. 2013. “Do information‐Rich Contexts Reduce Knowledge Inequalities? The Contextual Determinants of Political Knowledge in Europe.” Acta Politica 48, no. 2: 119–143. 10.1057/ap.2012.34.

[bjos70020-bib-0026] Fukkink, R. 2019. “Kansen Voor Kinderen in De Amsterdamse Kinderopvang.” In Gelijke Kansen in De Stad, edited by H. Van de Werfhorst and E. Van Hest , 23–35: Amster.

[bjos70020-bib-0027] Garg, R. , and S. Sengupta . 2019. ““When You Can Do it, Why Can’T I?”: Racial and Socioeconomic Differences in Family Technology Use and Non‐use.” Proceedings of the ACM on Human‐Computer Interaction 3, no. CSCW: 1–22: (CSCW). 10.1145/3359165.34322658

[bjos70020-bib-0028] Gesthuizen, M. , and P. Scheepers . 2010. “Educational Differences in Volunteering in Cross‐National Perspective.” Nonprofit and Voluntary Sector Quarterly 41, no. 1: 58–81. 10.1177/0899764010394203.

[bjos70020-bib-0029] Geven, S. , and D. Zwier . 2025. “Students’ Interactional Cultural Capital and Academic Performance in Test‐ and Teacher‐Based Assessments.” British Journal of Sociology 76, no. 3: 622–634. 10.1111/1468-4446.13199. 40055140 PMC12163557

[bjos70020-bib-0030] Granovetter, M. 1973. “The Strength of Weak Ties.” American Journal of Sociology 78, no. 6: 347–367. 10.1086/225469.

[bjos70020-bib-0031] Greenberg, J. , and R. M. Fernandez . 2016. “The Strength of Weak Ties in MBA Job Search: A Within–Person Test.” Sociological Science 3: 296–316. 10.15195/V3.A14.

[bjos70020-bib-0032] Grieco, M. 1987. Keeping it in the Family: Social Networks and Employment Chance. Cir.Nii.Ac.Jp. Tavistock. https://cir.nii.ac.jp/crid/1130282268814385792.

[bjos70020-bib-0033] Grönlund, K. , and H. Milner . 2006. “The Determinants of Political Knowledge in Comparative Perspective.” Scandinavian Political Studies 29, no. 4: 386–406. 10.1111/j.1467-9477.2006.00157.x.

[bjos70020-bib-0034] Haggerty, B. B. , H. Du , D. P. Kennedy , T. N. Bradbury , and B. R. Karney . 2022. “Stability and Change in Newlyweds’ Social Networks over the First Years of Marriage.” Psycnet.Apa.Org. 10.1037/fam0001016. PMC9942941 35862079

[bjos70020-bib-0035] Hakhverdian, A. , and Q. Mayne . 2012. “Institutional Trust, Education, and Corruption: A Micro‐Macro Interactive Approach.” Journal of Politics 74, no. 3: 739–750. 10.1017/S0022381612000412/SUPPL_FILE/SUP001.DOC.

[bjos70020-bib-0036] Hillygus, D. S. 2005. “The Missing Link: Exploring the Relationship Between Higher Education and Political Engagement.” Political Behavior 27, no. 1: 25–47. 10.1007/s11109-005-3075-8.

[bjos70020-bib-0037] House, J. , D. Umberson , and K. Landis . 1988. “Structures and Processes of Social Support.” Annual Review of Sociology 14, no. 1: 293–318. https://www.jstor.org/stable/2083320.

[bjos70020-bib-0038] Hummel, B. , M. A. Yerkes , and M. Bal . 2023. “Unprecedented Injustice’: Digitalisation and the Perceived Accessibility of Childcare Benefits.” Journal of Social Policy: 1–20. 10.1017/S0047279423000521.

[bjos70020-bib-0039] Jahani, E. , S. P. Fraiberger , M. Bailey , and D. Eckles . 2023. “Long Ties, Disruptive Life Events, and Economic Prosperity.” PNAS 120, no. 28: e2211062120. 10.1073/pnas.2211062120. 37410864 PMC10334764

[bjos70020-bib-0040] Jansen, T. , J. Rademakers , G. Waverijn , R. Verheij , R. Osborne , and M. Heijmans . 2018. “The Role of Health Literacy in Explaining the Association Between Educational Attainment and the Use of out‐of‐hours Primary Care Services in Chronically Ill People: A Survey Study.” BMC Health Services Research 18, no. 1: 394. 10.1186/s12913-018-3197-4. 29855365 PMC5984471

[bjos70020-bib-0041] Karoly, L. A. , and G. C. Gonzalez . 2018. “Early Care and Education for Children in Immigrant Families.” Future of Children 21, no. 1: 71–101. 10.1353/foc.2011.0005. 21465856

[bjos70020-bib-0042] Kingston, P. , R. Hubbard , B. Lapp , P. Schroeder , and J. Wilson . 2003. “Why Education Matters.” Sociology of Education 7, no. 1: 53–70. https://www.jstor.org/stable/3090261.

[bjos70020-bib-0043] Knijn, T. , and J. Lewis . 2017. “ECEC: Childcare Markets in the Netherlands and England.” In Public or Private Goods? Redefining Res Publica, edited by B. Unger , D. van der Linde , and M. Getzner , 150–172. Edward Elgar Publishing.

[bjos70020-bib-0044] Kohler, U. , F. Class , and T. Sawert . 2024. “Control Variable Selection in Applied Quantitative Sociology: A Critical Review.” European Sociological Review 40, no. 1: 173–186. 10.1093/ESR/JCAC078.

[bjos70020-bib-0045] Kohler, U. , and K. Karlson . 2022. “KHB: Stata Module to Decompose Total Effects Into Direct and Indirect via KHB‐Method.” Statistical Software Components S457215. https://EconPapers.repec.org/RePEc:boc:bocode:s457215.

[bjos70020-bib-0046] Kulin, J. , and I. Johansson Sevä . 2021. “Who Do You Trust? How Trust in Partial and Impartial Government Institutions Influences Climate Policy Attitudes.” Climate Policy 21, no. 1: 33–46. 10.1080/14693062.2020.1792822.

[bjos70020-bib-0047] Lammers, M. , and L. Kok . 2021. “Are Active Labor Market Policies (Cost‐)Effective in the Long Run? Evidence From the Netherlands.” Empirical Economics 60, no. 4: 1719–1746. 10.1007/S00181-019-01812-3/FIGURES/2.

[bjos70020-bib-0048] Lareau, A. 2015. “Cultural Knowledge and Social Inequality.” American Sociological Review 80, no. 1: 1–27. 10.1177/0003122414565814.

[bjos70020-bib-0049] Lareau, A. , and E. B. Weininger . 2003. “Cultural Capital in Educational Research: A Critical Assessment.” Theory and Society 32, no. 5: 567–606. 10.1023/b:ryso.0000004951.04408.b0.

[bjos70020-bib-0050] Lastikka, A. L. , and L. Lipponen . 2016. “Immigrant Parents’ Perspectives on Early Childhood Education and Care Practices in the Finnish Multicultural Context.” International Journal of Multicultural Education 18, no. 3: 75–94. 10.18251/ijme.v18i3.1221.

[bjos70020-bib-0051] Lin, N. 2003. “Social Networks and Status Attainment.” Annual Review of Sociology 25, no. 1: 467–487. 10.1146/ANNUREV.SOC.25.1.467.

[bjos70020-bib-0052] Madden, E. F. 2015. “Cultural Health Capital on the Margins: Cultural Resources for Navigating Healthcare in Communities With Limited Access.” Social Science & Medicine 133: 145–152. 10.1016/J.SOCSCIMED.2015.04.006. 25864151

[bjos70020-bib-0053] Martin, L. , L. Delaney , and O. Doyle . 2024. “Everyday Administrative Burdens and Inequality.” Public Administration Review 84, no. 4: 660–673. 10.1111/puar.13709.

[bjos70020-bib-0054] McArthur, M. , and G. Winkworth . 2017. “What Do We Know About the Social Networks of Single Parents Who Do Not Use Supportive Services?” Child & Family Social Work 22, no. 2: 638–647. 10.1111/CFS.12278.

[bjos70020-bib-0055] McPherson, M. , L. Smith‐Lovin , and J. Cook . 2001. “Birds of a Feather: Homophily in Social Networks.” Annual Review of Sociology 27, no. 1: 415–444. 10.1146/ANNUREV.SOC.27.1.415.

[bjos70020-bib-0056] Miller, P. , E. Votruba‐drzal , R. Levine , and A. S. Koury . 2014. “Immigrant Families’ Use of Early Childcare: Predictors of Care Type.” Early Childhood Research Quarterly 29, no. 4: 484–498. 10.1016/j.ecresq.2014.05.011.

[bjos70020-bib-0057] Moore, G. 1990. “Structural Determinants of Men’s and Women’s Personal Networks.” American Sociological Review 55, no. 5: 726. 10.2307/2095868.

[bjos70020-bib-0058] Nieuwenhuis, R. 2022. “No Activation Without Reconciliation? The Interplay Between ALMP and ECEC in Relation to Women’s Employment, Unemployment and Inactivity in 30 OECD Countries, 1985–2018.” Social Policy and Administration 56, no. 5: 808–826. 10.1111/SPOL.12806.

[bjos70020-bib-0059] Núñez, I. , and I. Livanos . 2010. “Higher Education and Unemployment in Europe: An Analysis of the Academic Subject and National Effects.” Higher Education 59, no. 4: 475–487. 10.1007/S10734-009-9260-7/TABLES/5.

[bjos70020-bib-0060] OECD . 2023. “Employment by Educational Level (Indicator).” 10.1787/19991266

[bjos70020-bib-0061] Paccoud, I. , J. Nazroo , and A. Leist . 2020. “A Bourdieusian Approach to Class‐Related Inequalities: The Role of Capitals and Capital Structure in the Utilisation of Healthcare Services in Later Life.” Wiley Online Library 42, no. 3: 510–525. 10.1111/1467-9566.13028. PMC7079030 31769062

[bjos70020-bib-0062] Pavolini, E. , and W. Van Lancker . 2018. “The Matthew Effect in Childcare Use: A Matter of Policies or Preferences?” Journal of European Public Policy 25, no. 6: 878–893. 10.1080/13501763.2017.1401108.

[bjos70020-bib-0063] Pena‐López, J. A. , and J. M. Sánchez‐Santos . 2017. “Individual Social Capital: Accessibility and Mobilization of Resources Embedded in Social Networks.” Social Networks 49: 1–11. 10.1016/J.SOCNET.2016.11.003.

[bjos70020-bib-0064] Rajkumar, K. , G. Saint‐Jacques , I. Bojinov , E. Brynjolfsson , and S. Aral . 2022. “A Causal Test of the Strength of Weak Ties.” Science 377, no. 6612: 1304–1310. 10.1126/science.abl4476. 36107999

[bjos70020-bib-0065] Renema, J. 2018. Immigrants’ Support for Welfare Spending. the Causes and Consequences of Welfare Usage and Welfare Knowledgeability. Radboud University.

[bjos70020-bib-0066] Roeters, A. , and F. Bucx . 2016. Beleidssignalement over Het Gebruik Van Kinderopvang Door Ouders Met Lagere Inkomens. Sociaal En Cultureel Planbureau.

[bjos70020-bib-0067] Roeters, A. , and F. Bucx . 2018. “Kijk Op Kinderopvang: Hoe Ouders Denken over De Betaalbaarheid, Toegankelijkheid En Kwaliteit Van Kinderopvang. Sociaal En Cultureel Planbureau.” Sociaal En Cultureel Planbureau.

[bjos70020-bib-0068] Rözer, J. J. , B. Hofstra , M. E. Brashears , and B. Volker . 2020. “Does Unemployment Lead to Isolation? The Consequences of Unemployment for Social Networks.” Social Networks 63: 100–111. 10.1016/J.SOCNET.2020.06.002.

[bjos70020-bib-0069] Seibel, V. 2021. “What Do Migrants Know About Their Childcare Rights? A First Exploration in West Germany.” Journal of International Migration and Integration 22, no. 3: 1181–1202. 10.1007/s12134-020-00791-0.

[bjos70020-bib-0070] Seibel, V. , and T. Hedegaard . 2017. “Migrants’ and Natives’ Attitudes to Formal Childcare in the Netherlands, Denmark and Germany.” Children and Youth Services Review 78: 112–121. 10.1016/j.childyouth.2017.05.017.

[bjos70020-bib-0071] Shahid, R. , M. Shoker , L. M. Chu , R. Frehlick , H. Ward , and P. Pahwa . 2022. “Impact of Low Health Literacy on Patients’ Health Outcomes: A Multicenter Cohort Study.” BMC Health Services Research 22, no. 1: 1148. 10.1186/s12913-022-08527-9. 36096793 PMC9465902

[bjos70020-bib-0072] Statistics Netherlands . 2021. “Recht Op En Gebruik Van Kinderopvangtoeslag, 2015‐2021.” https://www.cbs.nl/nl‐nl/maatwerk/2023/07/recht‐op‐en‐gebruik‐van‐kinderopvangtoeslag‐2015‐2021.

[bjos70020-bib-0073] Van der Waal, J. , W. de Koster , T. van Meurs , K. Noordzij , J. O. Groeniger , and J. Schaap . 2024. “Advancing Stratification Research by Measuring Non‐Declarative Cultural Capital: A National Population‐Based Study Combining IAT and Survey Data.” American Sociological Review 89, no. 4: 735–760. 10.1177/00031224241261603.

[bjos70020-bib-0074] Van Dam, C. , R. Van Aalst , and R. Leijten . 2020. Ongekend Onrecht. Parlementaire Ondervraging Kinderopvangtoeslag. https://www.njb.nl/media/4165/ongekend‐recht.pdf.

[bjos70020-bib-0075] Van Der Heide, I. , J. Wang , M. Droomers , P. Spreeuwenberg , J. Rademakers , and E. Uiters . 2013. “The Relationship Between Health, Education, and Health Literacy: Results From the Dutch Adult Literacy and Life Skills Survey.” Supplement, Journal of Health Communication 18, no. S1: 172–184. 10.1080/10810730.2013.825668. 24093354 PMC3814618

[bjos70020-bib-0076] Van Gameren, E. 2013. “The Role of Economic Incentives and Attitudes in Participation and Childcare Decisions.” Journal of Family and Economic Issues 34, no. 3: 296–313. 10.1007/s10834-012-9332-1.

[bjos70020-bib-0077] Vesely, C. K. 2013. “Early Childhood Research Quarterly Low‐Income African and Latina Immigrant Mothers’ Selection of Early Childhood Care and Education (ECCE): Considering the Complexity of Cultural and Structural Influences.” Early Childhood Research Quarterly 28, no. 3: 470–486. 10.1016/j.ecresq.2013.02.001.

[bjos70020-bib-0078] Waldfogel, J. 2006. “What Do Children Need?” Public Policy Research 13, no. 1: 26–34. 10.1111/j.1070-3535.2006.00417.x.

[bjos70020-bib-0079] Wang, W. , Y. Ma , T. Wu , Y. Dai , X. Chen , and L. A. Braunstein . 2019. “Containing Misinformation Spreading in Temporal Social Networks.” Chaos: An Interdisciplinary Journal of Nonlinear Science 29, no. 12: 123131. 10.1063/1.5114853. 31893637

[bjos70020-bib-0080] Welteke, C. , and K. Wrohlich . 2019. “Peer Effects in Parental Leave Decisions.” Labour Economics 57: 146–163. 10.1016/J.LABECO.2019.02.008.

[bjos70020-bib-0081] Yerkes, M. , and J. Javornik . 2019. “Creating Capabilities: Childcare Policies in Comparative Perspective.” Journal of European Social Policy 29, no. 4: 529–544. 10.1177/0958928718808421.

[bjos70020-bib-0082] Zamanbin, M. , M. Yerkes , and V. Seibel . 2025. “Between Benefits and Burdens: Navigating the Dutch Landscape of Childcare Allowances From a Migrant Perspective.” Social Policy and Administration: 1–14. 10.1111/SPOL.13142.

