# Supplementary material for: The Strength of Weak Ties? Understanding Educational Differences in Parents' Childcare Benefit Knowledge by Applying a Social Capital Approach
Source: Br J Sociol. 2025 Aug 5;76(5):1063–75. doi: 10.1111/1468-4446.70020 (PMC12668249; doi:10.1111/1468-4446.70020)
Supplement: Supplementary file 1 — Supporting Information S1 [file BJOS-76-1063-s001.rtf]

Table A. 1: Six scenarios of couples with varying employment status*
	Partner 1	Partner 2	Eligible for childcare benefits according to Dutch law (2022)	
Scenario 1	Permanent job	Unemployed for 12 months and following re-integration trajectory	Yes	
Scenario 2	Permanent job	Unemployed for 7 months and caring for sick mother	No	
Scenario 3	Permanent job	Part-time employed	Yes	
Scenario 4	Permanent job	Full-time study	Yes	
Scenario 5	Full-time study	Full-time study	Yes	
Scenario 6	Unemployed for 2 months	Unemployed for 2 months	Yes	
*Scenarios 1, 4, 5, and 6 referred to specific eligibility exceptions that also entitle non-working parents to receive childcare benefits (i.e., if they are following a re-integration trajectory, an educational program or have been unemployed for less than 3 months). Scenario 3 tested parents' knowledge about benefit eligibility based on variation in working hours (i.e., full-time versus part-time work). This scenario is particularly important in the Netherlands, as it has the highest part-time work rate across industrialized countries and 80% of mothers works part-time (Portegijs, 2022). Ultimately, the total amount of benefits parents receive is related to the amount of childcare hours eligible for the benefit, which is tied to the work hours of the parent working the fewest hours. Finally, scenario 2 tested whether parents are aware of their ineligibility for childcare benefits, namely, if one parent is taking care of a sick relative. 


Table A.2: Descriptives (Mean, SD, Min, Max) of all variables
Variable	Mean	Std. Dev.	Min.	Max.	
Childcare benefits knowledge
	0.39	0.26	0	1	
Educational level	 	 	 	 	
Low	0.	 	0	1	
Intermediate	0.37	 	0	1	
High	0.56	 	0	1	
Weak ties	 	 	 	 	
No information provision	0.66	 	0	1	
Information provision	0.24	 	0	1	
Don't know	0.10	 	0	1	
Strong ties	 	 	 	 	
No information provision	0.50	 	0	1	
Information provision	0.40	 	0	1	
Don't know	0.10	 	0	1	
Controls	 	 	 	 	
Age	38.98	6.57	22	67	
Female	0.57	 	0	1	
Employed	0.84	 	0	1	
Parents	0.87	 	0	1	
Married	0.61	 	0	1	
N	863	 	 	 	


Table A.3: Linear regressions: Impact of education and social capital on parents' knowledge about childcare benefits (including migration background and income as control)
	Model A	Model B	Model C	Model D	Model E	
Educational level						
Low	Ref. 	Ref.	Ref.	Ref.	Ref.	
Intermediate	0.059	0.038	0.040	0.038	0.072	
	(0.159)	(0.332)	(0.317)	(0.334)	(0.199)	
High	0.088*	0.062	0.065	0.063	0.105+	
	(0.036)	(0.118)	(0.105)	(0.112)	(0.059)	
Weak ties						
No information provision		Ref.		Ref.	Ref.	
Information provision		0.020		0.031	0.218*	
		(0.356)		(0.204)	(0.047)	
Don't know		-0.253***		-0.259***	-0.199*	
		(0.000)		(0.000)	(0.025)	
Strong ties						
No information provision			Ref.	Ref.	Ref.	
Information provision			-0.008	-0.021	-0.042	
			(0.706)	(0.352)	(0.653)	
Don't know			-0.262***	0.000	0.000	
			(0.000)	Omitted	Omitted	
Interactions						
Weak ties: Information provision						
   #Intermediate educated					-0.215+	
					(0.066)	
   #Highly educated					-0.190+	
					(0.092)	
Weak ties: Don't know						
   #Intermediate educated					-0.149	
					(0.121)	
   #Highly educated					0.008	
					(0.940)	
Strong ties: Information provision						
   #Intermediate educated					0.066	
					(0.513)	
   #Highly educated					0.001	
					(0.995)	
Strong ties: Don't know						
   #Intermediate educated					Omitted	
   #Highly educated					Omitted	
						
Controls						
Age	-0.008***	-0.007***	-0.007***	-0.007***	-0.007***	
	(0.000)	(0.000)	(0.000)	(0.000)	(0.000)	
Female	0.058**	0.052*	0.050*	0.051*	0.051*	
	(0.009)	(0.016)	(0.020)	(0.017)	(0.017)	
Employed	0.022	0.021	0.019	0.022	0.030	
	(0.469)	(0.465)	(0.502)	(0.451)	(0.300)	
Language spoken growing up (1= Dutch)	0.053+	0.053+	0.047	0.052+	0.057+	
	(0.093)	(0.081)	(0.115)	(0.087)	(0.065)	
Income (netto)	0.000	0.000	0.000	0.000	0.000	
	(0.130)	(0.276)	(0.267)	(0.289)	(0.422)	
parents	0.093**	0.091**	0.087**	0.088**	0.092**	
	(0.004)	(0.003)	(0.006)	(0.005)	(0.003)	
married	-0.014	-0.013	-0.014	-0.014	-0.013	
	(0.493)	(0.485)	(0.450)	(0.455)	(0.516)	
R²						
N	702	702	702	702	702	
p-values in parentheses
+ p<0.10, * p<0.05, ** p<0.01, *** p<0.001
